# Developmental Potential of Abnormally Fertilized Oocytes and the Associated Clinical Outcomes

**DOI:** 10.3389/fphys.2020.528424

**Published:** 2020-11-04

**Authors:** Xiao Chen, Shuai Shi, Jiating Mao, Libo Zou, Keda Yu

**Affiliations:** Reproductive Medicine Center, Jinhua People’s Hospital, Jinhua, China

**Keywords:** discarded embryo, blastocyst, monopronuclear, extending culture, pregnant rate

## Abstract

This study aims to investigate the embryo development potential of extending the culture of abnormally fertilized zygotes with no pronuclear (0PN), monopronuclear (1PN), and poor-quality day 3 embryos and to determine the associated clinical outcomes. This is a retrospective study performed between January 2014 and May 2018 at Jinhua People’s Hospital. The normal developed embryos and the abnormal 0PN, 1PN, and poor-quality day 3 embryos were cultured to day 5 or 6 for embryo transfer. Clinical outcomes resulting from abnormal embryos and normally developed embryos were compared. A total of 6466 embryos (1542 0PN, 852 1PN, and 4072 poor-quality day 3 embryos) from 831 treatment cycles were cultured to the blastocyst stage. The total blastulation rate was 17.3% (1121/6466) with 18.2% in 0PN, 26.1% in 1PN, and 15.2% in poor-quality day 3 embryos. The rate for good-quality blastocyst formation was 9.5% (616/6466) with 11.2% in 0PN group, 14.8% in 1PN group, and 7.8% in poor-quality day 3 embryos, respectively. Blastulation rates of 0PN and 1PN derived from intracytoplasmic sperm injection (ICSI) were significantly lower compared with the *in vitro* fertilization group. A total of 243 cycles were transferred with blastocysts originating from abnormal embryos, resulting in 109 (44.9%) clinical pregnancies and 19 (17.4%) miscarriages; in the control group, a total of 350 cycles resulted in 214 (61.1%) clinical pregnancies and 18 (8.4%) miscarriages. The live birth rate was significantly lower in the abnormal embryo group than that in the control group. Collectively, conventional *in vitro* fertilization derived 0PN and 1PN zygotes, not ICSI, together with day 3 embryos with poor quality, that were able to reach the blastocyst stage and produce a fair pregnancy rate and live birth rate.

## Introduction

Women have had greater access to contraceptive methods, professional education, and careers during the 20th century, which has resulted in women marrying and having children at later ages (Steiner and Jukic, 2016). Women face a gradual decrease in fertility after 35 years old, which is characterized by higher miscarriage rates and lower fecundability (Irani et al., 2020). This trend has been attributed to the rise in aneuploidy rates in these women (Franasiak et al., 2014). Those women are at high risk of treatment cancelation because of a lack of available embryos. Embryologists have established different assessment systems to evaluate the quality of embryos. The conventional method of embryo assessment for transfer is based on evaluating morphologic parameters during embryonic development. As frequent removal of embryos from the incubator for assessment may lead to unexpected pH and temperature shifts in the embryo culture medium, the conventional assessment of embryo morphology is limited to once a day at set time points (Desai et al., 2014). However, the development of embryos is dynamic and static assessment of embryonic growth can limit their ability to discern differences between embryos at similar stages. Recently, the time-lapse imaging and monitoring of the *in vitro* fertilization (IVF) application has enabled more detailed analysis on the developmental kinetics of embryos (Kovacs et al., 2019).

Fertilization is defined by the confirmation of the second polar body and two pronuclei (2PN) (ALPHA Scientists In Reproductive Medicine, and ESHRE Special Interest Group Embryology, 2011). Zygotes showing other than 2PN are usually considered as abnormally fertilized, and usually discarded because of the high risk of chromosomal defect. It seems that the assessment of fertilization is straightforward, nevertheless, some confounding aspects exist. Firstly, the appropriate assessment timepoint for zygotes originating from IVF and intracytoplasmic sperm injection (ICSI) are different. Development of zygotes arising from IVF is 1 h later than those from ICSI. A recent study performed with time-lapse technology indicated that developmental kinetics of embryos are different between IVF and ICSI embryos, especially at the early stage of embryo development, until the five cell stage (Bodri et al., 2015). Secondly, the time for pronuclear formation and syngamy is different. Staessen et al. (1993) evaluated 312 1PN zygotes twice (at 16–18 h and at 20–24 h after insemination) and found that 25% of the zygotes manifested the second pronucleus 4-6 h later (van der Heijden et al., 2009). Furthermore, polar bodies can fragment and disappear before fertilization checks (ALPHA Scientists In Reproductive Medicine, and ESHRE Special Interest Group Embryology, 2011). These confounding factors provided potential explanations as to why abnormal pronuclear-stage embryos are not necessarily genetically defective (van der Heijden et al., 2009). Furthermore, normal pregnancies derived from monopronulcear (1PN) zygotes have been reported (Staessen et al., 1993; Reichman et al., 2010). However, due to the limited number of embryos presented in these studies, whether normal pregnancies could be reliably derived from 1PN zygotes is still questionable. Morphological assessments of cleavage-stage embryos provide essential information to predict embryo quality and implantation potential. The morphological parameters considered include blastomere number, evenness of cell size, and amount of cellular fragmentation. Cell number reflects the developmental stage of the embryo, and has been agreed as 4 cells on day 2 and 8 cells on day 3 post-insemination. Both slower and faster cleaved embryos have a reduced implantation rate. Unevenly sized blastomeres and the amount of fragmentation were associated with a negative impact on pregnancy outcomes. However, this evaluation system is not perfect. Morphologically perfect embryos do not always successfully implant in the uterus, conversely, embryos of suboptimal appearance could produce healthy babies. Furthermore, authors reported that extending the culture of poor-quality day 2 embryos could improve assisted reproductive technology (ART) outcomes (Poulain et al., 2014; Kaartinen et al., 2015).

Upon our review of the existing literature, we found that few studies examined the developmental fate and suitability of 0PN, 1PN, and poor-quality day 3 embryos simultaneously. Therefore, the aim of this study was to investigate embryo development potentials of the extending culture of abnormally fertilized zygotes with no pronuclear (0PN) and monopronuclear (1PN) and poor-quality day 3 embryos and to determine the associated clinical outcomes.

## Materials and Methods

### Study Group

This retrospective study was conducted at Jinhua People’s Hospital of China between January 2014 and May 2018. The study was approved by the Ethics Committee of Jinhua People’s Hospital and informed consent has been obtained from all patients in the study. A total of 1181 consecutive patients subjected to IVF/ICSI were included in the present study. The inclusion criteria for these patients were: (1) No uterine malformations, uterine fibroids, or adenomyosis; (2) No history of habitual abortion; and (3) No history of hereditary or family diseases. Among the 1181 patients, 588 patients failed to have embryo implantation. The remaining 593 patients were further classified into two groups. For the group A patients, a total of 243 patients (33.4 ± 4.2 years old) were implanted with abnormal embryos, and the criteria for abnormal embryos were: (i) abnormally fertilized zygotes with 0PN and 1PN; and (ii) poor-quality day 3 embryos, which were not suitable for transfer nor cryopreservation. For the group B patients (the control group), a total of 350 patients (32.7 ± 4.5 years old) were implanted with normally developed embryos, and the criteria for the normally developed embryos were: zygotes displayed successful extrusion of the second polar body and two even PN (2PN), and embryos were classified as grade I-III.

### Ovarian Stimulation and Fertilization Methods

On day 3 of the menstrual period, a basic evaluation was conducted by ultrasound examination. Medication was then initiated with recombinant follicle-stimulating hormone (rFSH), Gonal-F (EMD Serono), in which younger patients (<35 years old) were advised to take two ampoules (150 IU) of rFSH daily, and older patients (≥35 years old) were advised to take three ampoules (225 IU) of rFSH daily. Similarly, the dose was fixed for the first four days of stimulation, and after four consecutive days of medication, transvaginal ultrasound B examination was then carried out to monitor the development of follicles. The dose of rFSH was optimally adjusted based on the ultrasound B results for the number and size of developing follicles. The GnRH antagonist, cetrorelix, was next administered daily by s.c. injection (0.25 mg/d) in the morning (8:00-12:00 AM) from day 5 of the stimulation cycle to the day of human chorionic gonadotropin (HCG) administration. rFSH and GnRH antagonist were administered continuously until three follicles reached ≥18 mm. HCG (10,000 IU, EMD Serono) was then administrated. Oocytes were retrieved 34–36 h after the administration of HCG by transvaginal aspiration under ultrasound guidance. Oocytes were collected in a MOPS/HEPES-buffered medium (MHM, Irvine Scientific, United States). After the retrieval, insemination was performed with the use of either conventional IVF or ICSI. For patients in the ICSI group, insemination was performed by using ICSI. At 3–4 h after oocyte retrieval, the cumulus–oocyte complex (COC) was stripped by using hyaluronidase. Only matured oocytes were inseminated. For patients in the conventional IVF group, insemination was performed by conventional IVF. At 2 h after oocyte retrieval, collected COCs were inseminated for another 2 h, at a concentration of 100,000 motile sperm/ml. Inseminated COCs were cultured overnight in culture medium. The details for the ICSI and IVF treatment for the patients were summarized in Supplementary Table S1. A fertilization check was performed 16–18 h after insemination. Normally fertilized zygotes displayed successful extrusion of the second polar body and 2PN. Abnormal zygotes showed 0PN or 1PN. All the normal and abnormal zygotes were cultured under the same conditions until day 5 or 6. The blastulation rates and the good-quality blastocyst formation rates were analyzed.

### Embryo Culture, Quality Assessment, and Embryo Transfer

Embryos (single culture) were cultured with 25 μl G-1 Plus microdroplet (Vitrolife company, Sweden) at a pH of 7.2 in a humidified incubator at 37°C and 6% CO_2_. Fragmentation rate, blastomere size, and cell number of day 3 cleavage embryos were analyzed. The quality assessment was analyzed by the same embryologist. Based on the amount of fragmentation, all embryos were scored as four grades: (i) grade I embryo with fragmentation ≤ 5%, (ii) grade II embryo with 5–20% fragmentation, (iii) grade III embryo with 20–50% fragmentation, and (iv) grade IV embryo with fragmentation ≤50%. Embryo score was increased by one grade in either of the following situations: unequal blastomere size and blastomere number <6. Embryos were directly scored as grade IV if blastomere number ≤4. Good-quality embryos on day 3 were defined as ≥6 evenly sized cells with ≤20% fragmentation (Grade I and II), and grade I, II, and III embryos were classified as available embryos. Embryos scored as grade IV were considered as poor-quality embryos and normally were discarded. In this study, the meant-to-be-discarded grade IV embryos on day 3 were transferred to balanced G-2 Plus microdroplet (Vitrolife Company, Sweden) for extended culture until day 6.

The quality assessment of blastocysts was based on Gardner classification. Based on this scoring scheme, we defined good-quality blastocysts as expansion grade ≥ 3, inner cell mass grade ≤ B, and trophectoderm grade ≤ B on day 5. For the day 6 blastocysts, the good-quality criteria were expansion grade ≥ 4, inner cell mass grade ≤ B, and trophectoderm grade ≤ B. Blastocysts that scored equal or better than 3CC on day 5 and 4CC on day 6 were classified as available embryos. After fresh blastocyst was transferred, the extra available embryos were all cryopreserved. Embryo transfer was performed 5 or 6 days post-insemination based on the availability of the patients. Embryo transfer was performed with a Wallace catheter (Marlow/Cooper Surgical, Shelton, CT, United States). The number of embryos transferred to a given patient was determined by the number and quality of embryos she had available, the patient’s age, and her prior clinical history.

### Follow-Up for Pregnancy Rates

The pregnancy rates were followed up until birth in all the patients (group A and group B) from the study. Clinical pregnancy was defined as the presence of a gestational sac with a fetal heartbeat on ultrasound examination 28 days after embryo transfer. A loss of pregnancy before the 20th week of gestation was referred to as miscarriage. Ectopic pregnancy was defined as a pregnancy in which implantation had occurred outside of the uterine cavity. Live birth rate was defined as the number of deliveries with at least one live born baby resulting from one initiated or aspirated ART cycle according to The International Glossary on Infertility and Fertility Care, 2017 (Zegers-Hochschild et al., 2017). The live birth rate was compared between group A and group B patients.

### Statistical Analysis

Data analysis was performed using SPSS 16.0 software (IBM, SPSS statistics). The data were normally distributed as determined by the Kolmogorov–Smirnov test. All the data were presented either as mean ± standard deviation or percentage (%), as appropriate. Significant difference between different groups for continuous data was analyzed by unpaired Student’s *t*-test or one-way ANOVA followed by Bonferroni’s *post hoc* test, as appropriate. Categorical data were analyzed using Chi-square test. A *P*-value less than 0.05 was considered statistically significant.

## Results

**FIGURE 1:**
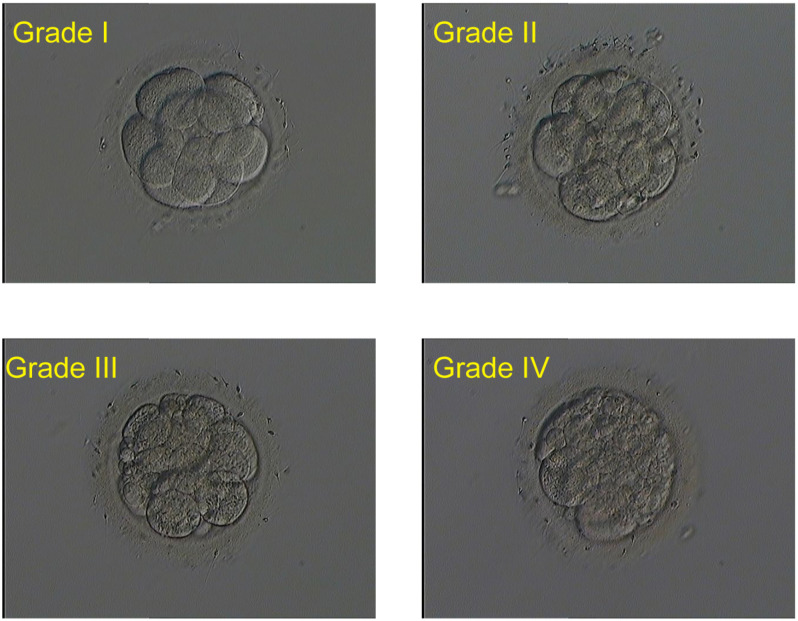
Embryos at day 3 in different grades. Magnification at 200x.

During the study period, 6466 embryos (1542 0PN, 852 1PN, and 4072 poor-quality day 3 embryos) from 831 treatment cycles from patients with abnormally fertilized zygotes were cultured to the blastocyst stage. The images of the day 3 embryos of different grades were shown in Figure 1. Of the total cohort of abnormal embryos, the blastulation rate was 17.3% (1121/6466) and good-quality blastocyst formation rate was 9.5% (616/6466). Both blastulation and good-quality blastocyst rates were significantly higher in the 1PN group when compared to the 0PN group, regardless of the embryo quality assessed on day 3 (*P* < 0.001; Tables 1, 2). The poor-quality day 3 embryo group (2PN) had a higher blastulation and good-quality blastocyst formation rates than that from the 0PN and 1PN groups in the same quality grade (*P* < 0.001; Tables 1, 2). Further comparison revealed that Grade I-II embryos presented the highest blastulation and good-quality blastocyst rate when compared with grade III and IV in both 0PN and 1PN zygotes (*P* < 0.001; Tables 1, 2).

**TABLE 1:** Total blastulation rate of abnormal fertilized (0PN and 1PN) and poor-quality day 3 embryos (2PN).

Day 3 embryo quality	0PN	1PN	2PN	
I-II	46.2% (174/377)^a^	57.0% (126/221)^a^	–	0.0104
III	17.8% (38/214)^a^	36.7% (59/161)^a^	–	<0.001
IV	7.3% (69/951)^a^	7.9% (37/470)^a^	15.2% (618/4072)	<0.001
Total	18.2% (281/1542)	26.1% (222/852)	15.2% (618/4072)	<0.001

*^*a*^*P* < 0.001 comparison among I-II, III, and IV groups. Significant differences between different groups were analyzed by Chi-square test. 0PN, no pronuclear; 1PN, monopronuclear; 2PN, two pronuclei; NS, non-significant.*

**TABLE 2:** Good-quality blastocyst rate of abnormal fertilized (0PN and 1PN) and poor-quality day 3 embryos (2PN).

Day 3 embryo quality	0PN	1PN	2PN	
I-II	35.0% (132/377)^a^	40.3% (89/221)^a^	–	NS
III	6.5% (14/214)^a^	11.8% (19/161)^a^	–	NS
IV	2.7% (26/951)^a^	3.8% (18/470)^a^	7.8% (318/4072)	<0.001
Total	11.2% (172/1542)	14.8% (126/852)	7.8% (318/4072)	<0.001

*^*a*^*P* < 0.001 comparison among I-II, III, and IV groups. Significant differences between different groups were analyzed by Chi-square test. 0PN, no pronuclear; 1PN, monopronuclear; 2PN, two pronuclei; NS, non-significant.*

There were no statistical differences when comparing blastulation and good-quality blastocyst rate between women less than 35 years old with those above 35 (*P* > 0.05; Tables 3, 4). Insemination methods significantly influenced the rates of blastocyst formation in 0PN and 1PN groups, but not in the 2PN group. Blastulation rates of 0PN and 1PN derived from ICSI were significantly lower compared with the IVF group (*P* < 0.5; Table 3). Good-quality blastocyst rates of 0PN and 1PN derived from the ICSI group were not significantly different from the IVF group (*P* > 0.05; Table 4).

**TABLE 3:** Prognostic factors associated with blastulation rate when Grade IV day 3 embryos.

Variables	0PN	1PN	2PN
**Female age**			
=35	7.9% (62/788)	8.6% (31/362)	15.6% (508/3258)
>35	4.3% (7/163)	5.6% (6/108)	13.5% (110/814)
*P*-value	NS	NS	NS
**Insemination methods**			
IVF	7.8% (69/881)	8.8% (37/421)	16.0% (510/3178)
ICSI	0 (0/70)	0 (0/49)	12.2% (108/885)
*P*-value	0.0071	0.0232	NS

*Significant differences between different groups were analyzed by Chi-square test. 0PN, no pronuclear; 1PN, monopronuclear; 2PN, two pronuclei; ICSI, intracytoplasmic sperm injection; IVF, *in vitro* fertilization; NS, non-significant.*

**TABLE 4:** Prognostic factors associated with good-quality blastocyst rate when Grade IV day 3 embryos.

Variables	0PN	1PN	2PN
**Female age**			
=35	3.1% (24/788)	4.4% (16/362)	8.1% (263/3258)
>35	1.2% (2/163)	1.9% (2/108)	6.8% (55/814)
*P*-value	NS	NS	NS
**Insemination methods**			
IVF	2.9% (26/881)	4.3% (18/421)	8.2% (262/3187)
ICSI	0 (0/70)	0 (0/49)	6.3% (56/885)
*P*-value	NS	NS	NS

*Significant differences between different groups were analyzed by Chi-square test. 0PN, no pronuclear; 1PN, monopronuclear; 2PN, two pronuclei,; ICSI, intracytoplasmic sperm injection; IVF, *in vitro* fertilization; NS, non-significant.*

During the study period, a total of 243 cycles were transferred with blastocysts arising from abnormal embryos (group A), and 350 cycles transferred with normally developed blastocysts were recruited as control (group B). Implantation rate (32.3% vs. 42.4%, *P* = 0.002, Table 5) and clinical pregnancy rate (44.9% vs. 61.1%, *P* < 0.001, Table 5) were significantly lower in women transferred with blastocysts arising from abnormal embryos, when compared with the control group. Miscarriage rate was significantly higher in the abnormal embryo group than control (17.4% vs. 8.4%, *P* = 0.02, Table 5), and the ectopic pregnancy rate was no different between the two groups (2.8% vs. 2.3%, *P* > 0.05, Table 3). Further analysis of live birth rate from the pregnancy patients showed that the live birth rate was significantly lower in the abnormal embryo group than in the control group (27.5% vs. 57.0%, *P* < 0.001, Table 5). Furthermore, we stratified the group A patients based on the quality of the zygotes. As shown in Table 6, 114 patients received single embryo transfer and 129 patients received double embryo transfer. Among the patients who received single embryo transfer, there was no significant difference in the clinical outcomes among the 0PN, 1PN, and 2PN groups (*P* > 0.05, Table 6). Among the patients who received double embryo transfer, the 2PN group showed the highest implantation rate and clinical pregnancy rate, but it was not statistically significant (*P* > 0.05, Table 6); in addition, there was no statistical significance in the other clinical outcomes among these groups (*P* > 0.05, Table 6).

**TABLE 5:** Comparison of blastocyst transferred cycles resulting in clinical outcomes according to whether they are originating from discarded or normal embryos.

	Group A	Group B	*P*-value
Cycles (*n*)	243	350	–
Age (year)	33.4 ± 4.2	32.7 ± 4.5	NS
No. of embryos transferred	1.5 ± 0.5	1.6 ± 0.5	NS
Implantation rate (%)	32.3 (120/372)	42.4 (238/562)	0.002
Clinical pregnancy rate (%)	44.9 (109/243)	61.1 (214/350)	<0.001
Miscarriage (%)	17.4 (19/109)	8.4 (18/214)	0.02
Ectopic pregnancy rate (%)	2.8 (3/109)	2.3 (5/214)	NS
Live birth rate (%)	27.5 (30/109)	57.0 (122/214)	<0.001

*Group A, cycles transferred with blastocysts arising from discarded embryos; Group B, cycles transferred with blastocysts arising from normal developing embryos. Significant difference was analyzed by unpaired Student’s t-test or Chi-square test. NS, non-significant.*

**TABLE 6:** Comparison of blastocyst transferred cycles resulting in clinical outcomes originated from discarded embryos.

	Single embryo transfer			Double embryo transfer					
		
	0PN	1PN	2PN	0PN, 0PN	0PN, 1PN	0PN, 2PN	1PN, 1PN	1PN, 2PN	2PN, 2PN
Cycles (*n*)	28	35	51	12	8	19	5	23	62
Age (year)	33.3 ± 4.7	33.8 ± 4.4	32.6 ± 4.9	31.67 ± 4.1	34.0 ± 3.2	34.4 ± 2.8	33.20 ± 4.6	34.3 ± 4.1	33.6 ± 3.5
Implantation rate (%)	25.0 (7/28)	28.6 (10/35)	31.4 (16/51)	20.8 (5/24)	25.0 (4/16)	31.6 (12/38)	30.0 (3/10)	37.0 (17/46)	37.1 (46/124)
Clinical pregnancy rate (%)	25.0 (7/28)	28.6 (10/35)	31.4 (16/51)	41.7 (5/12)	50.0 (4/8)	52.6 (10/19)	40.0% (2/5)	60.9 (14/23)	66.1 (41/62)
Miscarriage (%)	28.6 (2/7)	10.0 (1/10)	18.8 (3/16)	20.0 (1/5)	25.0 (1/4)	20.0 (2/10)	0.0 (0/2)	14.3 (2/14)	17.1 (7/41)
Ectopic pregnancy rate (%)	0 (0/7)	10.0 (1/10)	0 (0/16)	0 (0/5)	0 (0/4)	0 (0/10)	0 (0/2)	7.1 (1/14)	2.4 (1/41)
Live birth rate (%)	14.3 (1/7)	30.0 (3/10)	25.0 (4/16)	40.0 (2/5)	25.0 (1/4)	30.0 (3/10)	0 (0/2)	21.4 (3/14)	31.7 (13/41)

## Discussion

With the advancement of ART, embryologists have developed mature systems to mimic *in vivo* growth environments for embryos. However, *in vitro* culture is still a suboptimal condition compared to physical state, which compels the embryo to undergo physiological adaptations, such as delayed cell division, reduction in oxidation metabolism, and increase in lactate production. Morphology analysis provides limited information on those dynamic changes in developing embryos. Studies have reported that abnormally fertilized oocytes can be rescued and result in healthy live births (Itoi et al., 2015; Bradley et al., 2017; Capalbo et al., 2017), but these studies were still at the preliminary stages. In the study from Itoi et al. (2015), only 33 transfer cycle blastocysts derived from 1PN embryos were performed and resulted in nine deliveries. Bradley et al. (2017) reported a transfer of 20 IVF and six ICSI 1PN-derived blastocysts. The study from Capalbo et al. (2017) performed genetic analysis (PGT-A) cycles and found that from 1PN-derived blastocysts three were diploid, nine were haploid, and one was triploid. Though the practice of extending the culture of poor-quality day 2 embryos to improve ART outcomes, whether the authors analyzed blastocyst formation from day-2 poor quality embryos or day-3 was not clearly stated (Yao et al., 2016; Sallem et al., 2018). Our data showed that 17.3% of abnormal embryos reached the blastocyst stage and 9.5% were good-quality blastocyst. A total of 243 cycles were transferred with blastocysts arising from abnormal embryos, resulting in 109 (44.9%) clinical pregnancies. Such findings suggested that abnormally fertilized 0PN and 1PN zygotes, together with poor-quality day 3 embryos, should not be discarded because of the potential clinical application.

Zygotes with an abnormal number and shape of pronuclei at the fertilization check in IVF treatments are routinely discarded owing to the increased risk of ploidy abnormalities. The morphological features of zygotes are related to the time post-fertilization; zygotes’ scoring must be performed within a fixed time period, usually 16–18 h after insemination, based on the static assessment of embryonic growth. However, a recent study performed with time-lapse technology has demonstrated that a good percentage of oocytes (around 15–20%) have shown a 2PN fading/disappearing before the 16 h post injection (Campbell et al., 2013). In a clinical study, 22,308 zygotes were analyzed during this time period; 8.0% were already in syngamy (ALPHA Scientists In Reproductive Medicine, and ESHRE Special Interest Group Embryology, 2011). 0PN zygotes observed at fertilization check still have as adequate a potential to develop to blastocysts and implant into the uterus as with 2PN embryos. In the present study, 18.2% of 0PN developed into the blastocyst stage and 11.2% were in good quality.

The European Society of Human Reproduction and Embryology recommended that individual laboratories should make their own decision on whether to discard or continue culture *in vitro* of 1PN zygotes (ALPHA Scientists In Reproductive Medicine, and ESHRE Special Interest Group Embryology, 2011). Furthermore, previous studies demonstrated that a considerable proportion of 1PN zygotes are diploid and healthy babies have been obtained (Staessen and Van Steirteghem, 1997; Mateo et al., 2013, 2017). In this study, we observed 26.1% of 1PN zygotes reached the blastocyst stage, and the good-quality blastocyst rates were 14.8%. van der Heijden et al. (2009) reported that the rate of diploid chromosomes in 1PN zygotes were 86.7% in IVF and 30.3% in ICSI. In our study, blastulation rates of 0PN and 1PN derived from ICSI were significantly lower compared with the IVF group. These results recommended that 0PN and 1PN embryos derived from IVF are available for extending culture and may lead to an increase in the chance of pregnancy in patients seeking ART treatments. However, ICSI-derived 0PN and 1PN should be discarded because of the poor clinical application.

The morphology of day 3 embryos was a good prognosis for blastocyst development and clinical outcomes. The correlation between embryo morphology and chromosome abnormalities has been extensively studied (Munne, 2006; Gianaroli et al., 2007; Munne et al., 2007). Dysmorphisms, such as fragmentation, multinucleation, and evenness, is associated with an increased risk of post-meiotic abnormalities and decreased embryo developmental potential (Munne, 2006). Our study supported this conclusion; as shown in Table 1, good-quality embryos (Grade I-II) derived from 0PN and 1PN presented with significantly higher blastocyst formation and good-quality rates than that of Grade III and IV. Munne et al. (2007) reviewed more than 6000 embryos in patients younger than 35 years old and found that only 44% of the best morphology embryos were euploid. The worst morphology group still had 30% normal embryos (Munne, 2006). In the present study, we observed 15.2% Grade IV 2PN embryos developed into the blastocyst stage, and 7.8% were good-quality blastocysts.

This study has several limitations. The present study is a single center study, and multiple center studies may further verify our findings. The follow-up study was limited to the live birth rates, and the assessment of the health status of the newborns may require further follow-up. The present study lacks the genetic analysis of preimplantation genetic testing for aneuploidy (PGT-A) before transfer, as our laboratory is still in the second generation of IVF. Future studies may perform PGT-A1 before the embryo transfer to improve the ART outcomes.

Embryologists and clinicians hesitated over using abnormal embryos because of the increased risk of genetic abnormalities, which may produce high rates of miscarriage and birth defects. The implantation and clinical pregnancy rates obtained by blastocysts derived from abnormal embryos were lower than that in the normal control group, and the miscarriage rate was higher, but the live birth rate was lower in the abnormal embryo group. This is particularly important for older women with poor ovarian preservation. In the present study, there is no significant difference in the clinical outcomes among different groups stratified by zygotes quality, though the 2PN, 2PN group showed the highest implantation rate and clinical pregnancy rate. This may be due to the small sample size in each subgroup, and future studies should increase the sample size to confirm associations between the quality of zygotes and clinical outcomes. The data regarding the health status of children from abnormally fertilized zygotes using ART has not been reported. However, we have to be cautious about the health of newborns, as studies have found that singleton ICSI and IVF 5-year-olds from normally fertilized zygotes are more likely to need health care resources than naturally conceived children (Bonduelle et al., 2005). Thus, the health of the children in the present study should be carefully followed in the future, in order to further confirm the safety of this strategy.

## Conclusion

An acceptable percentage of blastocyst development can be obtained using 0PN, 1PN, and poor-quality day 3 embryos, which may lead to an increased chance of pregnancy in patients receiving ART treatment.

## Data Availability Statement

The raw data supporting the conclusions of this article will be made available by the authors, without undue reservation, to any qualified researcher.

## Ethics Statement

The studies involving human participants were reviewed and approved by the study was approved by the Ethics Committee of Jinhua People’s Hospital. The patients/participants provided their written informed consent to participate in this study.

## Author Contributions

XC and KY designed the study, collected and processed the samples, performed the statistical analysis, and wrote the manuscript. SS and JM collected and processed the samples and participated in experiments. LZ was involved with the study design and revised the statistical analysis and the manuscript. All the authors read and approved the final manuscript.

## Conflict of Interest

The authors declare that the research was conducted in the absence of any commercial or financial relationships that could be construed as a potential conflict of interest.

## Abbreviations

0PN: no pronuclear
1PN: monopronuclear
ART: assisted reproductive technology
COS: controlled ovarian stimulation
hCG: human chorionic gonadotrophin
ICSI: intracytoplasmic sperm injection
IVF: *in vitro* fertilization.
