# Supplementary material for: Developmental Potential of Abnormally Fertilized Oocytes and the Associated Clinical Outcomes
Source: Front Physiol. 2020 Nov 4;11:528424. doi: 10.3389/fphys.2020.528424 (PMC7672117; doi:10.3389/fphys.2020.528424)
Supplement: Supplementary file 1 [file Table_1.docx]

Supplemental Table S1. Treatment details for the ICSI/IVF procedures

| Patients | ICSI (number of patients) | IVF (number of patients) |
| --- | --- | --- |
| Patients without embryo implantation | 83 | 505 |
| Group A patients with embryo implantation) | 32 | 211 |
| Group B patients with embryo implantation | 50 | 300 |

ICSI = intracytoplasmic sperm injection, IVF = in vitro fertilization
